# Supplementary material for: N-cadherin crosstalk with integrin weakens the molecular clutch in response to surface viscosity
Source: Nat Commun. 2024 Oct 12;15:8824. doi: 10.1038/s41467-024-53107-6 (PMC11479646; doi:10.1038/s41467-024-53107-6)
Supplement: Supplementary file 1 — Supplementary Information [file 41467_2024_53107_MOESM1_ESM.pdf]

N-cadherin crosstalk with integrin weakens the molecular clutch in response to surface viscosity

Eva Barcelona-Estaje[1], Mariana A.G. Oliva[1], Finlay Cunniffe[1], Aleixandre Rodrigo-Navarro[1], Paul Genever[2], Matthew J Dalby[1], Pere Roca-Cusachs[3,4*], Marco Cantini[1*], Manuel Salmeron-Sanchez[1*]

[1]Centre for the Cellular Microenvironment, Advanced Research Centre, Glasgow, UK
[2]Department of Biology, University of York, York, UK
[3]Institute for Bioengineering of Catalonia (IBEC), the Barcelona Institute of Technology (BIST), Barcelona, Spain
[4]University of Barcelona, Barcelona, Spain

*corresponding authors:
proca@ibecbarcelona.eu,
Marco.Cantini@glasgow.ac.uk,
Manuel.Salmeron-Sanchez@glasgow.ac.uk

Supplementary information

Supplementary Fig.1: hMSCs area with different concentrations of RGD

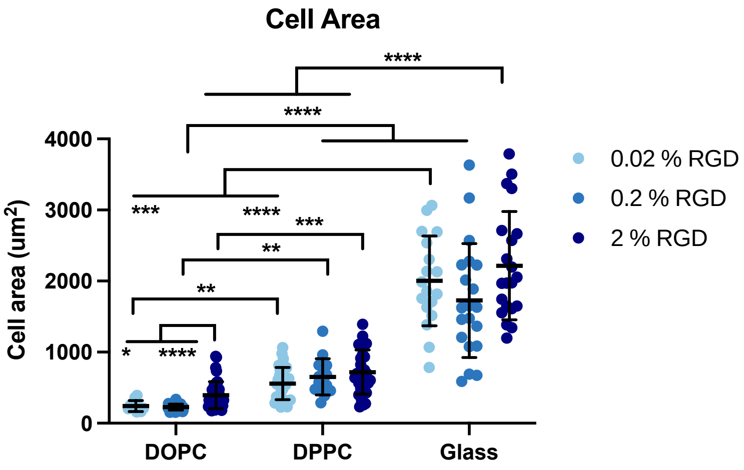

*Supplementary Figure 1: hMSCs area on SLBs and glass surfaces functionalized with different concentrations of RGD (in DOPC samples from left to right n=10, 36 and 40, in DPPC samples 34, 15 and 31, and Glass samples 18, 20 and 21) n represent cells. Statistical significance was determined using D'Agostino Pearson normality test, followed by a Kruskal–Wallis multiple-comparison test. All data are presented as mean values +/- SD.*P ≤ 0.05, **P ≤ 0.01, ***P ≤ 0.001, ****P ≤ 0.0001.*

Supplementary Fig.2: SLBs functionalized with FITC-RGD and Cy3-HAVDI

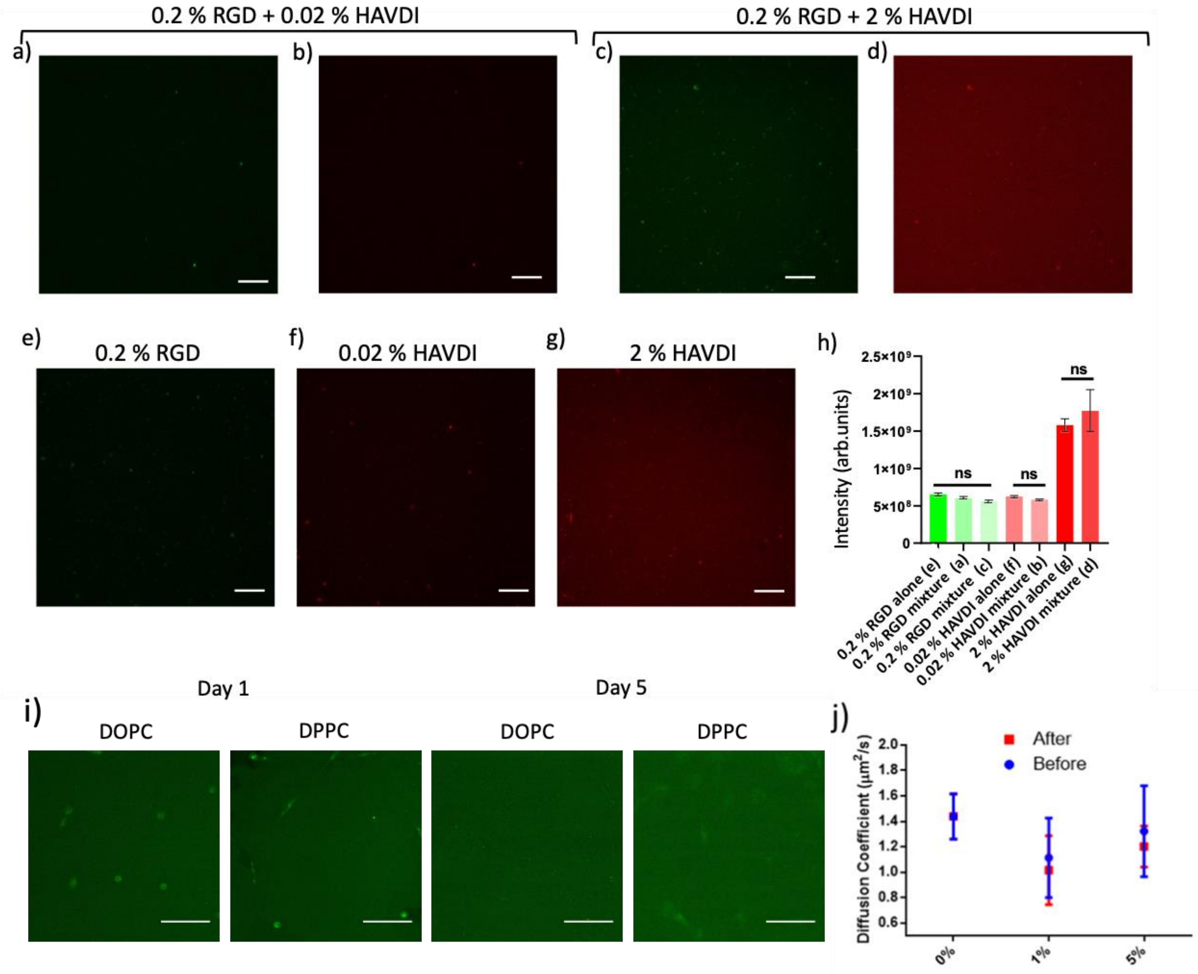

*Supplementary Figure 2: Fluorescence imaging of the functionalization of DOPC SLBs with FITC-RGD (green) and Cy-3-HAVDI (red). a,b) FITC-RGD (a) and Cy3-HAVDI (b) images of a bilayer functionalized with 0.2 % RGD and 0.02 % HAVDI. c,d) FITC-RGD (c) and Cy3-HAVDI (d) images of a bilayer functionalized with 0.2 % RGD and 2 % HAVDI. e) FITC-RGD image of a SLB functionalised with 0.2% RGD alone. f,g) Cy3-HAVDI image of a SLB functionalised with 0.02% (f) or 2 % (g) HAVDI alone. h) intensity of all the previous bilayers (n=15 in all conditions) n represents images of the bilayer. i) Representative images of RGD-functionalised DOPC and DPPC bilayers with 0.1 mol% BODIPY-functionalized lipids after 1 or 5 days of cell culture. j) Diffusion coefficient for the DOPC bilayer without functionalisation or with 1% or 5% of functionalisation, measured before or after the addition of neutravidin to the bilayer using fluorescence correlation spectroscopy as in Bennett et al.[1] Scale bar: 50 µm (n=15 for all conditions). Graph h shows the intensity of the ligands in the different bilayers. *P ≤ 0.05, **P ≤ 0.01, ***P ≤ 0.001, ****P ≤ 0.0001.*

Supplementary Fig. 3: N-Cadherin adhesion is involved in the changes observer in hMSCs

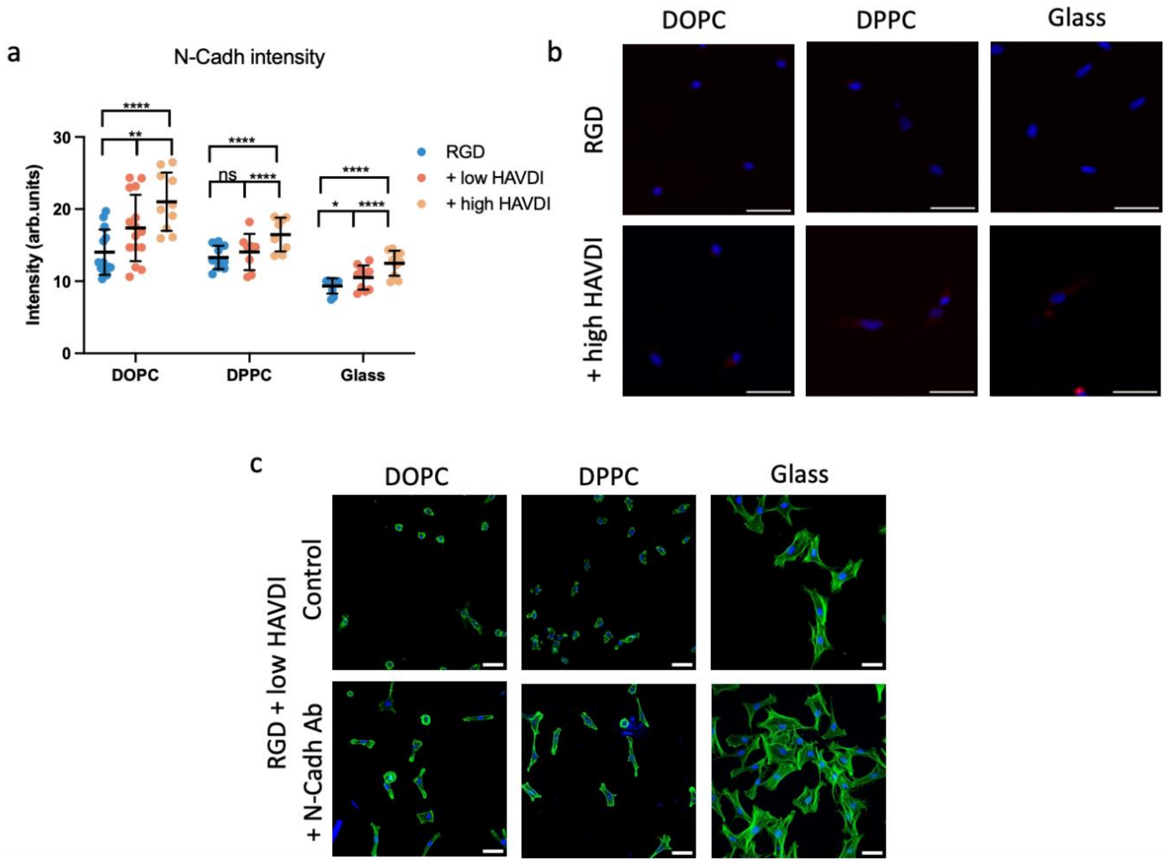

*Supplementary Figure 3: a) N-Cadherin intensity measured by immunostaining of hMSCs seeded on surfaces with RGD or RGD plus low or high HAVDI after 24 h of cell culture (from left to right n=15, 15, 10, 10, 8, 8, 9, 10, 10) in all conditions) n represent cells. b) Representative images of hMSCs seeded on surfaces with RGD or RGD plus high HAVDI stained for N-Cadherin (red) and DAPI (blue) after 24 h of cell culture. (scale bar= 50 µm) c) Representative images of hMSCs (with or without blocking their N-cadherins) seeded on surfaces with RGD plus low HAVDI stained for actin (green) and DAPI (blue) after 24 h of cell culture (scale bar= 100 µm). Statistical significance was determined using D'Agostino Pearson normality test, followed by a Kruskal−Wallis multiple-comparison test. All data are presented as mean values +/- SD. *P ≤ 0.05, **P ≤ 0.01, ***P ≤ 0.001, ****P ≤ 0.0001.*

Supplementary Fig. 4: Cell area and av clustering of hMSCs seeded on surfaces with varying concentrations of RGD and HAVDI

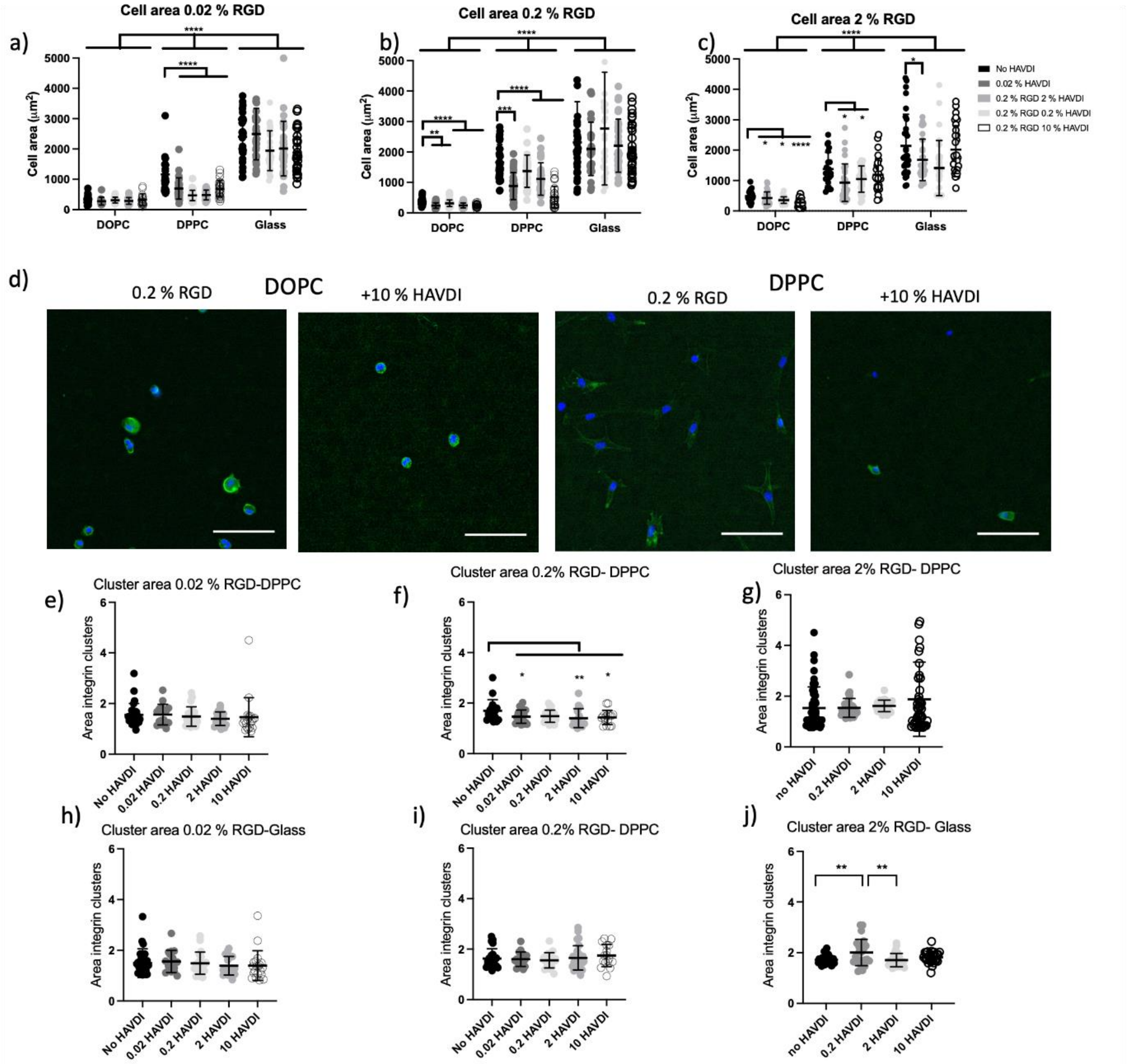

Supplementary Figure 4: a), b) and c) show cell area of hMSCs seeded on surfaces with 0.02 %, 0.2 % and 2 % of RGD respectively and varying concentrations of HAVDI. (n in graph a from left to right=26, 13, 13, 17, 16, 22, 30, 30, 22, 24, 29, 30, 30, 29, 30, n in graph b from left ro right= 29, 30, 30, 29, 24, 30, 30, 27, 27, 30, 30, 30, 30, 30, 31, n in graph c from left to right= 30, 30, 29, 30, 21, 30, 30, 30, 30, 30, 30, 30) Statistical significance was determined using D'Agostino Pearson normality test, followed by a Kruskal–Wallis multiple-comparison test. N represent cells. All data are presented as mean values +/- SD.d) shows representative images of cells on DOPC and DPPC without or with 10 % HAVDI. Scale bar in all the images: 100 mm. Cells are stained for DAPI (blue) and actin (green). Integrin avclusters formed by hMSCs after 24 h of cell culture on DPPC (e,f,g) and Glass (h,I,j) after 24 h of cell culture. RGD concentration was kept constant (0.02 % for e and h, 0.2 % for f and i and 2 % for g and j) and varying concentrations of HAVDI were added. (n in graph e from left to right=28, 16, 20, 19, 20, n in graph f from left to right= 17, 19, 21, 18,16, n in graph g from left to right= 50, 29, 29, 50, n in graph h from left to right= 27, 17, 20, 18, 20, n in graph i from left to right= 22, 18, 18, 27, 16, n in graph j from left to right= 28, 23, 28, 27) N represent cells. Statistical significance was determined using D'Agostino Pearson normality test, followed by a Kruskal–Wallis multiple-comparison test. All data are presented as mean values +/- SD. *P ≤ 0.05, **P ≤ 0.01, ***P ≤ 0.001, ****P ≤ 0.0001.

Supplementary Fig. 5: YAP translocation to the nucleus of hMSCs seeded on surfaces with different concentrations of RGD

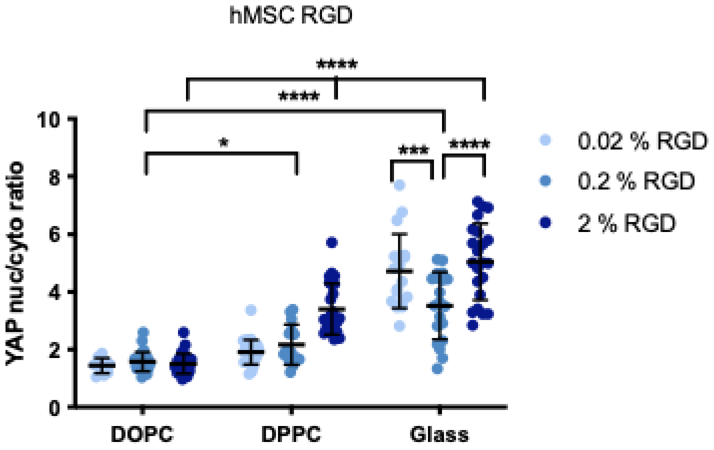

*Supplementary Figure 5: YAP translocation to the nucleus of hMSCs seeded con SLBs and glass surfaces with different concentrations of RGD for 24 h (DOPC from left to right n= 11, 36, 45, DPPC n= 34, 15, 25, and glass n= 18,20, 22) n represent cells. Statistical significance was determined using D'Agostino Pearson normality test, followed by a Kruskal–Wallis multiple-comparison test. All data are presented as mean values +/- SD. *P ≤ 0.05, **P ≤ 0.01, ***P ≤ 0.001, ****P ≤ 0.0001.*

Supplementary Fig. 6: MIIA and vinculin colocalization

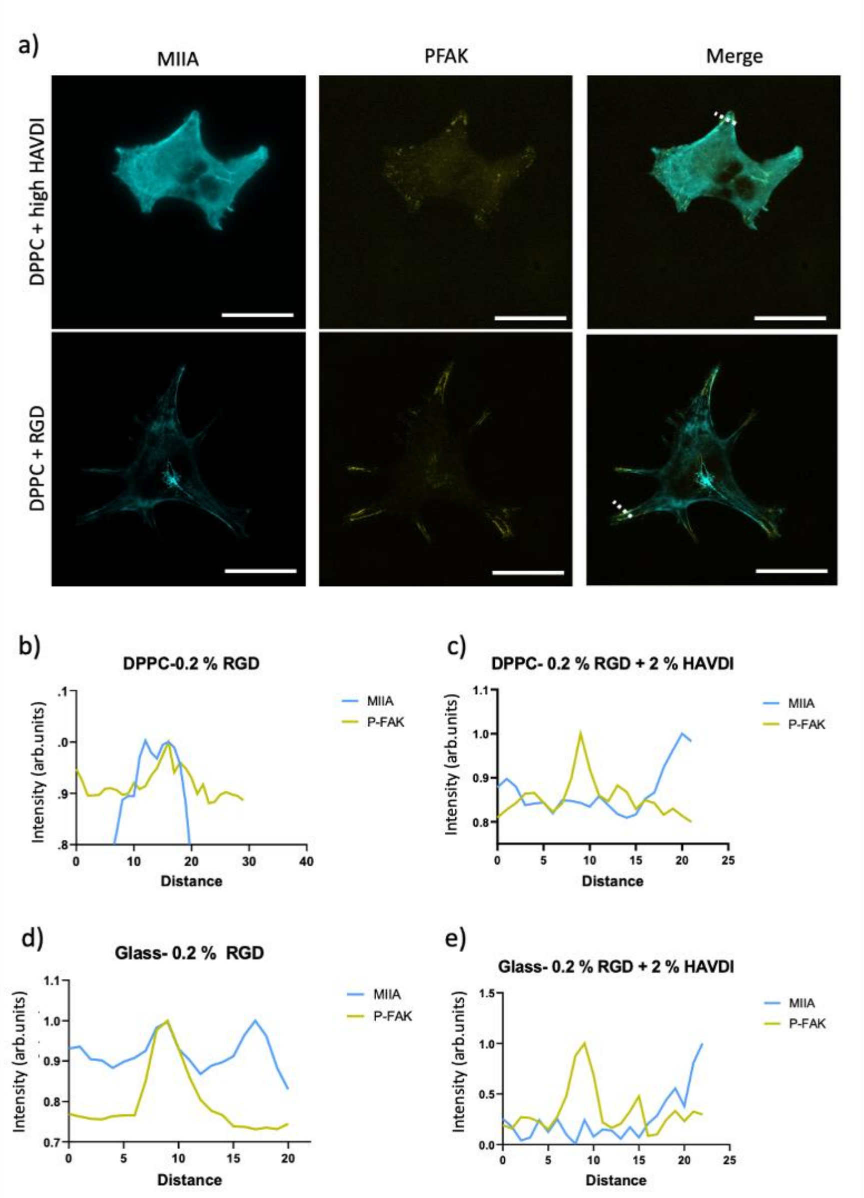

Supplementary Figure 6: Co-localization of MIIA with P-FAK in hMSCs. a) Representative images of hMSCs seeded on DPPC for 24 h stained for MIIA and P-FAK. The white line represents where the intensity profiles below have been taken. b, c, d and e are intensity profiles of FA sites of hMSCs stained for MIIA (blue) and P-FAK (yellow). Scale bar= 50 µm.

Supplementary Fig. 7: Early hMSCs differentiation markers

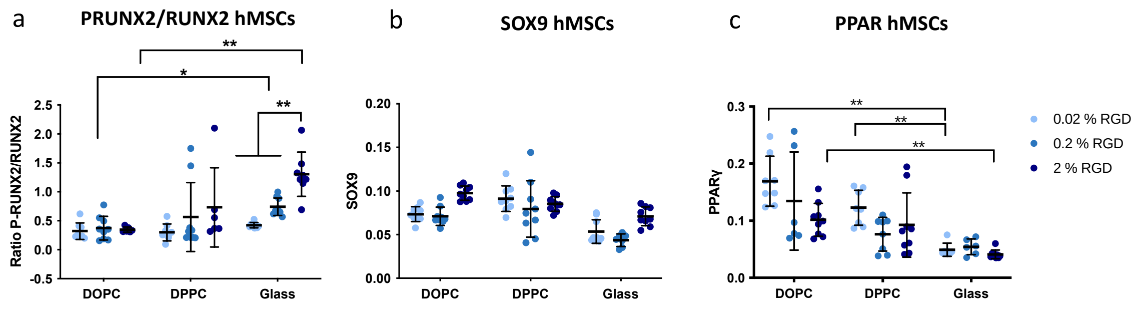

*Supplementary Figure 7: Early differentiation hMSCs marker expression measured by ICW for a) osteogenesis (7 days) (from left to right DOPC n=9,5,7; DPPC=9,8,8; glass=9,9,7(b) chondrogenesis (5 days) (from left to right DOPC n=9,9,9; DPPC= 9,9 9; glass= 9,9,7) and c) adipogenesis (5 days) (from left to right DOPC n=8,7,9; DPPC= 8,8,8; glass=7,6,9). N represents areas in the in-cell western wells. Statistical significance was determined using D'Agostino Pearson normality test, followed by a Kruskal–Wallis multiple-comparison test. All data are presented as mean values +/- SD. *P ≤ 0.05, **P ≤ 0.01, ***P ≤ 0.001, ****P ≤ 0.0001.*

Supplementary Fig. 8: Number of FAs of hMSCs

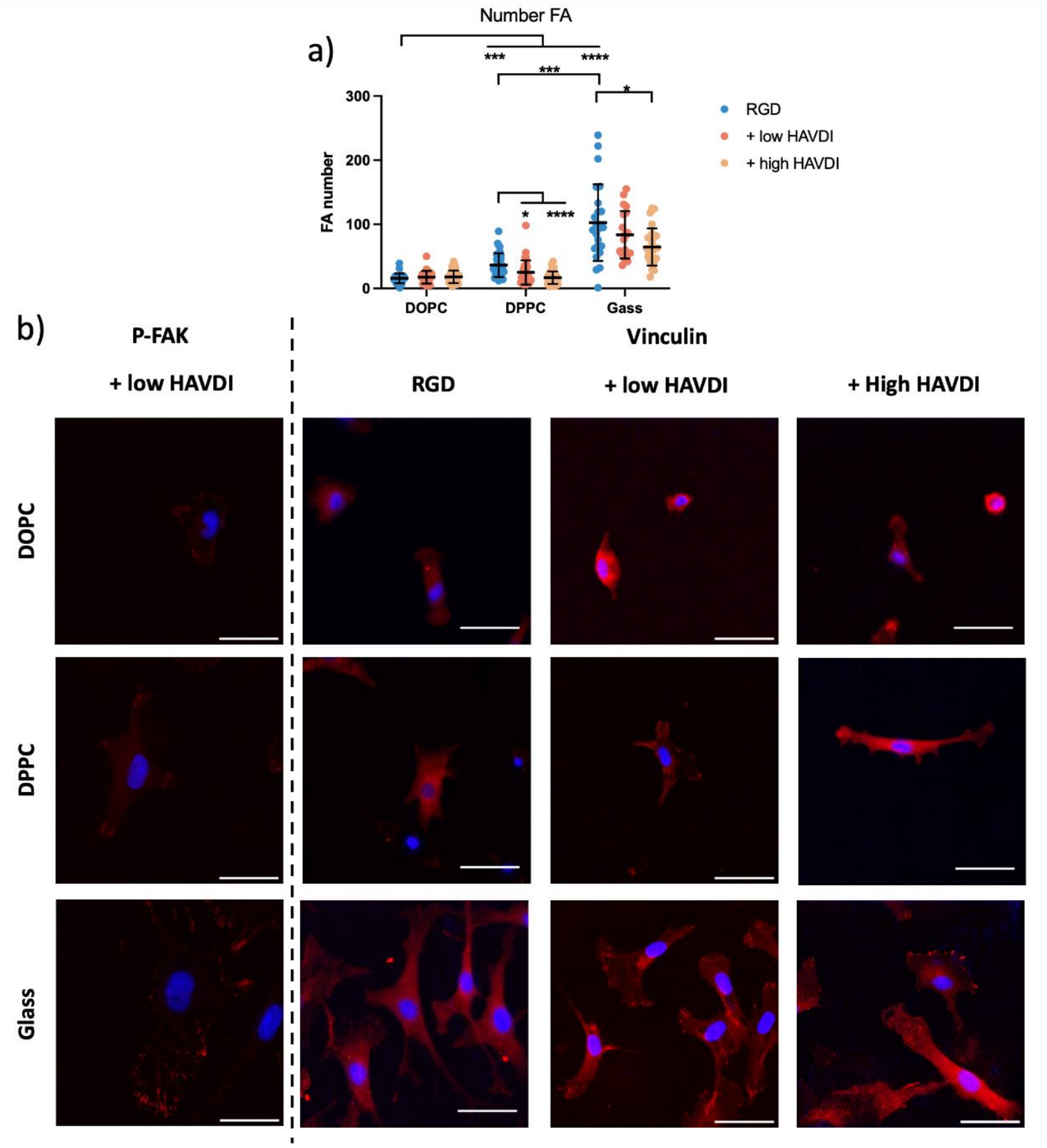

*Supplementary Figure 8: a) Number of FAs of hMSCs seeded on SLBs or glass surfaces functionalized with RGD or RGD plus low or high HAVDI for 24 h measured by vinculin immunostaining. (From left to right DOPC n=25,24,32; DPPC=35,34,51; glass=25,21,27). In all graphs n represent cells. *P ≤ 0.05, **P ≤ 0.01, ***P ≤ 0.001, ****P ≤ 0.0001. b) Representative images of focal adhesions of hMSCs stained for P-FAK seeded on SLBs (P-FAK= red; DAPI= blue) and glass with 0.2 % RGD or 0.2 % RGD plus low HAVDI and hMSCs seeded on SLBs and glass with 0.2 % RGD or 0.2 % RGD plus low or high HAVDI (P-FAK= red; DAPI= blue). Scale bar for all images = 50 µm. Statistical significance was determined using D'Agostino Pearson normality test, followed by a mixed effects analysis. All data are presented as mean values +/- SD.*

Supplementary Fig. 9: Area, Focal adhesions, YAP and early differentiation of Y201 cells

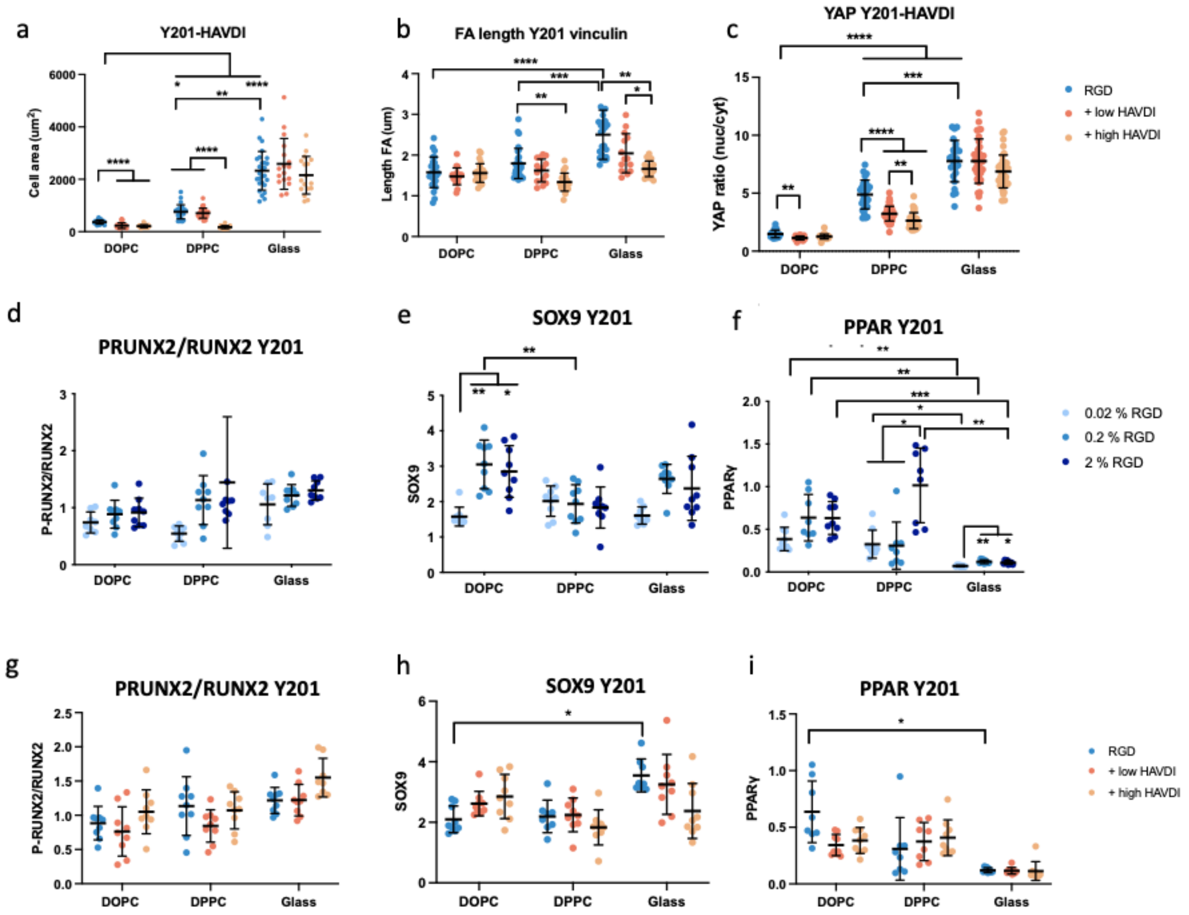

*Supplementary Figure 9: Area (a) (from left to right DOPC n=23, 22, 22; DPPC n=25, 27, 17; glass n=27, 17, 17), focal adhesion length (b) (from left to right DOPC n=26, 19, 26; DPPC n=25, 14, 20; glass=25, 14, 19) and YAP translocation to the nucleus (c) (from left to right DOPC n=20, 22, 29; DPPC n=29, 46, 34; glass n=26, 36, 38) of Y201 cells seeded on surfaces functionalized with RGD and RGD plus low or high HAVDI after 24 h, 24 h and 3 days respectively. In all graphs n represents cells. d) and g) show early osteogenic expression of Y201 cells seeded on surfaces functionalized with different concentrations of RGD or RGD plus low or high HAVDI respectively. e) and h) show early chondrogenic expression of Y201 cells seeded on surfaces functionalized with different concentrations of RGD or RGD plus low or high HAVDI respectively. f) and i) show early adipogenic expression of Y201 cells seeded on surfaces functionalized with different concentrations of RGD or RGD plus low or high HAVDI respectively (n=9 in all differentiation graphs and conditions). In all graphs n represents measured areas in the in-cell wells. Statistical significance was determined using D'Agostino Pearson normality test, followed by a mixed effects analysis. All data are presented as mean values +/- SD. *P ≤ 0.05, **P ≤ 0.01, ***P ≤ 0.001, ****P ≤ 0.0001.*

Supplementary Fig. 10: N-cadherin expression in talin overexpressed Y201 cells and focal adhesion formation in Jasplakinolide treated Y201 seeded on RGD and RGD plus low or high HAVDI

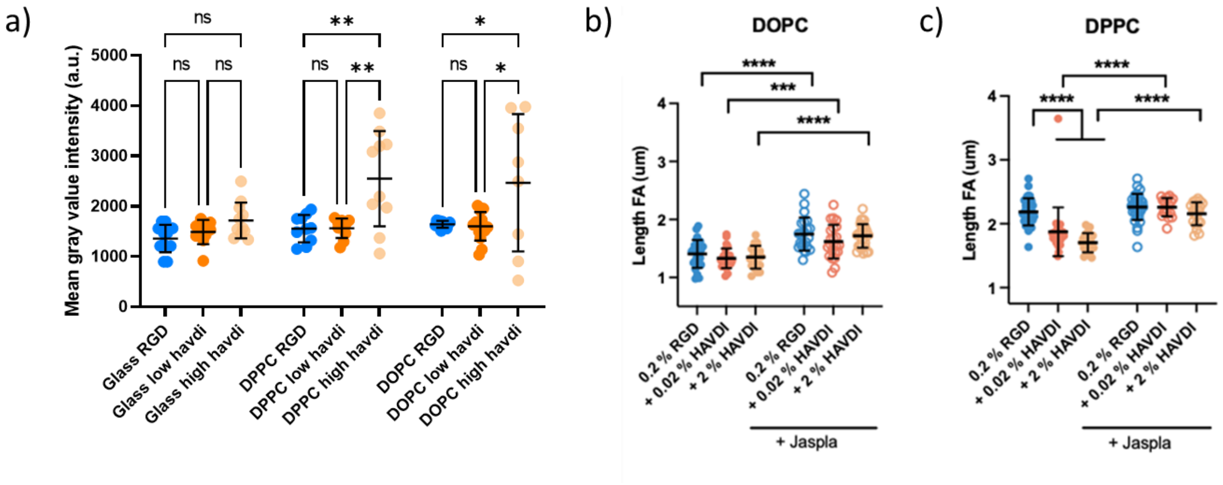

*Supplementary Figure 10: a) N-Cadherin intensity measured by immunostaining of talin overexpressed Y201 cells seeded on surfaces with RGD or RGD plus low or high HAVDI after 24 h of cell culture (from left to right Glass n=13, 10, 11; DPPC n=10, 10, 11; DOPC n=11, 14, 8). Statistical significance was determined using an Anderson-Darling normality test followed by an Ordinary one way-ANOVA. b,c) FA adhesion size of Y201 cells treated with Jasplakinolide for 1 h, seeded on DOPC (b) (from left to right n=26, 26, 20, 22, 23, 28) and DPPC (c) (from left to right n=24, 26, 21, 34, 19, 20). FA size measured by vinculin staining after 1 day of culture. Statistical significance was determined using D'Agostino Pearson normality test, followed by a Kruskal–Wallis multiple-comparison test. In all graphs n represent cells. All data are presented as mean values ± SD. *p ≤ 0.05, **p ≤ 0.01, ***p ≤ 0.001, ****p ≤ 0.0001.*

Supplementary Fig. 11: Focal adhesions in the presence of dorsal N-cadherin engagement

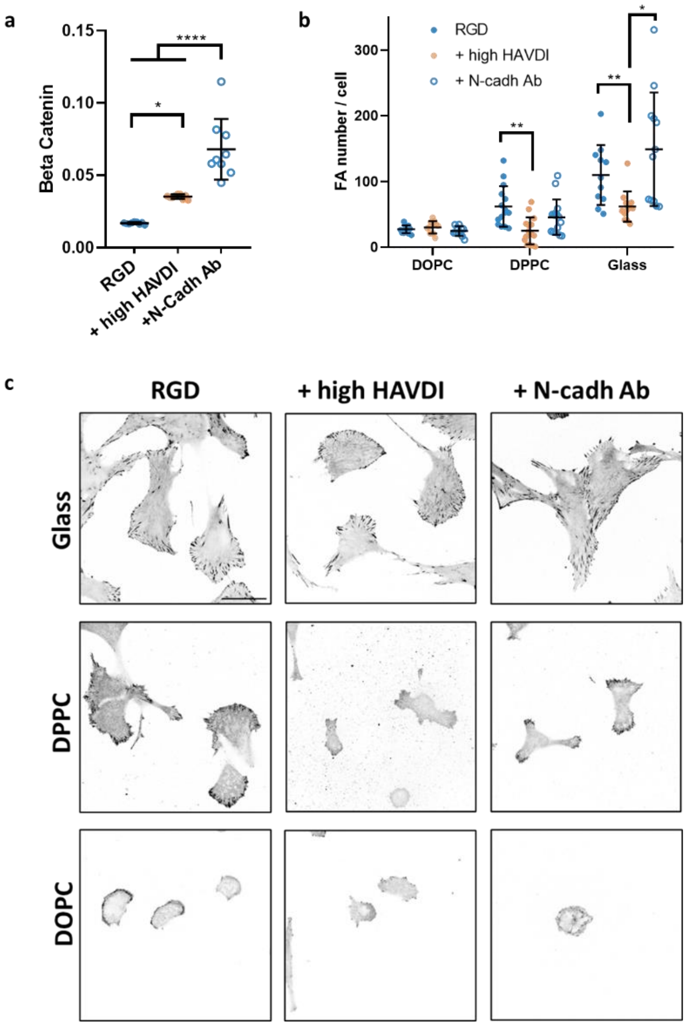

*Supplementary Figure 11: a) β-catenin expression, measured via In-Cell Western, of Y201 cells on RGD-functionalised glass; N-cadherin engagement was either promoted dorsally via an N-cadherin antibody or ventrally via addition of HAVDI on the surface, n=9 where n represent individual glass surfaces. Statistical significance was determined using an Anderson Darling normality test, followed by an Ordinary One-Way Anova with multiple comparisons. b) FA number of Y201 cells on DOPC, DPPC and glass functionalised with RGD, RGD+HAVDI, or RGD plus dorsal N-cadherin engagement (from left to right DOPC n=12, 11, 12; DPPC n=15 in all conditions; Glass n=11, 12, 13). In graph b, n represents individual cells. c) Representative vinculin staining after 1 day of culture. Scale bar = 50µm. Statistical significance was determined using a D'Agostino Pearson normality test, followed by a Kruskal-Walis multiple-comparisons test. All data are presented as mean values ± SD. *p ≤ 0.05, **p ≤ 0.01, ***p ≤ 0.001, ****p ≤ 0.0001.*

Supplementary Fig. 12: SCFS measurements of Y201 and hMSCs seeded on surfaces with different RGD concentrations and hMSCs seeded on surfaces with RGD or RGD plus low and high HAVDI

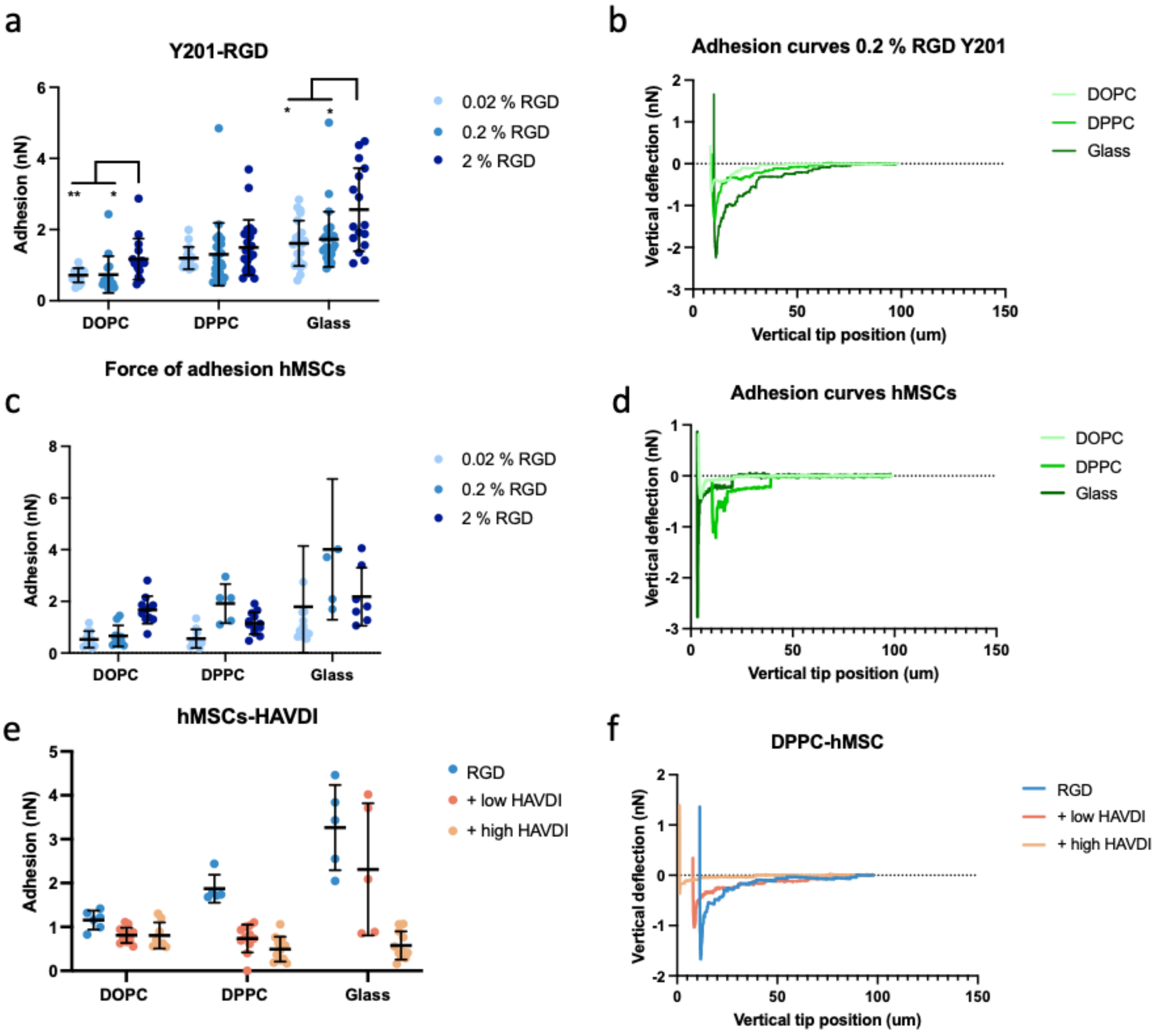

*Supplementary Figure 12: a) SCFS values of Y201 cells in contact for 30 seconds with surfaces functionalized with different concentrations of RGD (from left to right DOPC n=15, 16, 16; DPPC n=17, 24, 22; Glass n=22, 29, 16), see b for representative curves. c) SCFS values of hMSCs cells in contact for 30 seconds with surfaces functionalized with different concentrations of RGD (from left to right DOPC n=12, 10, 11; DPPC n=10, 5, 12; Glass n=12, 5, 7), see d for representative curves. e) SCFS values of Y201 cells in contact for 30 seconds with surfaces functionalized with different with RGD or RGD plus low or high HAVDI (from left to right DOPC n=10, 12, 9; DPPC n=5, 11, 11; Glass n=5, 5, 11), see f for representative curves. In all graphs n represents adhesion events. Statistical significance was determined using D'Agostino Pearson normality test, followed by a mixed effects analysis. All data are presented as mean values +/- SD. *P ≤ 0.05, **P ≤ 0.01, ***P ≤ 0.001, ****P ≤ 0.0001.*

Supplementary Fig. 13: Actin flow in the presence of dorsal N-cadherin engagement

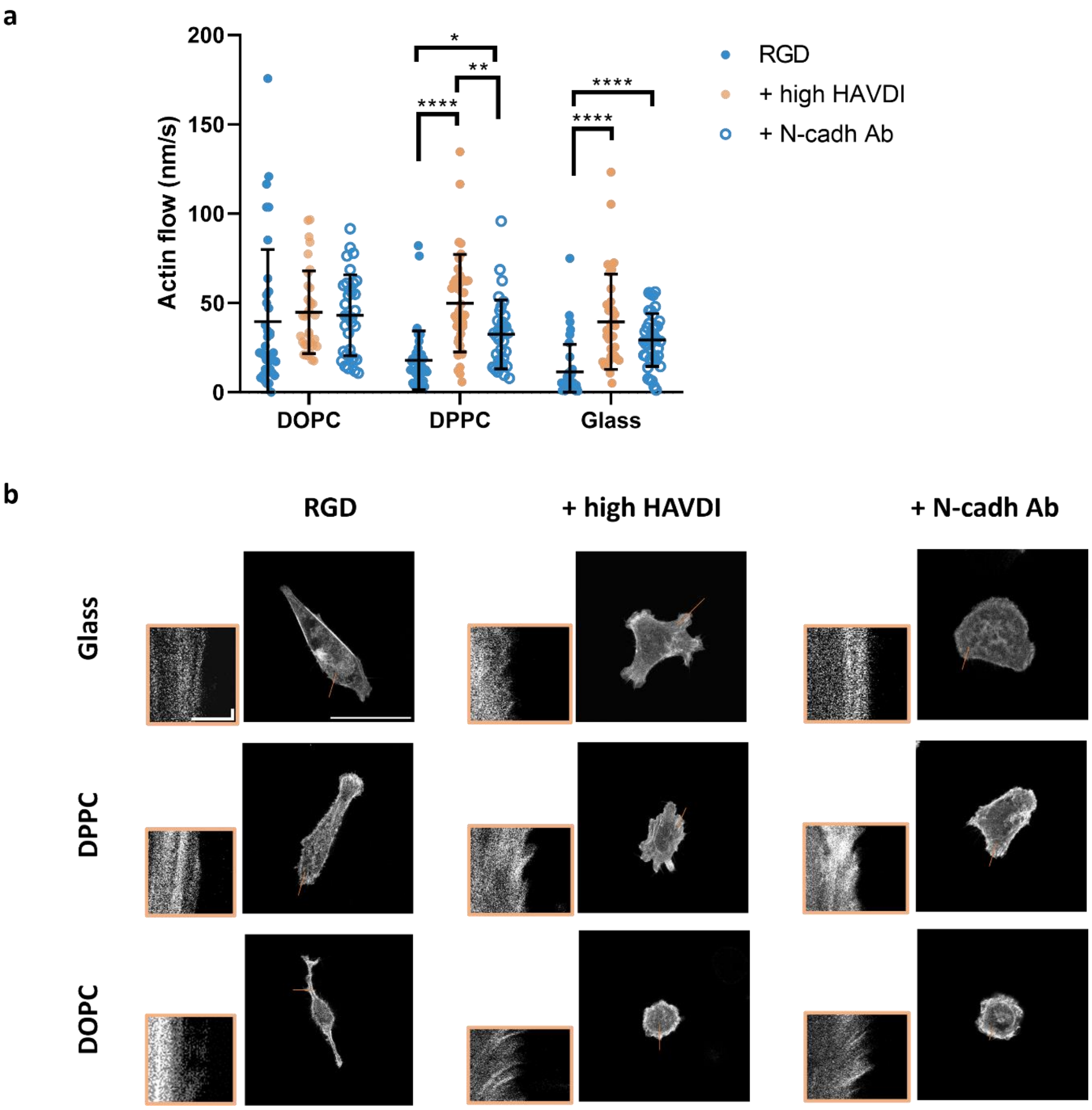

*Supplementary Figure 13: a) Actin flow of Y201 cells on DOPC, DPPC and glass functionalised with RGD, RGD+HAVDI, or RGD plus dorsal N-cadherin engagement (from left to right n=36, 34, 33, 40, 39, 33, 38, 33, 39. In all conditions n represent kymographs measured in 6-7 cells). b) Representative images of cells transfected with LifeAct and their corresponding kymographs. Scale bars: cell images = 50 µm, kymographs=5 µm horizontal and 30 s vertical. Statistical significance was determined using a D'Agostino Pearson normality test, followed by a Kruskal-Walis multiple-comparisons test. All data are presented as mean values ± SD. *p ≤ 0.05, **p ≤ 0.01, ***p ≤ 0.001, ****p ≤ 0.0001.*

Supplementary Fig. 14: Actin flow in vinculin overexpressed Y201 cells

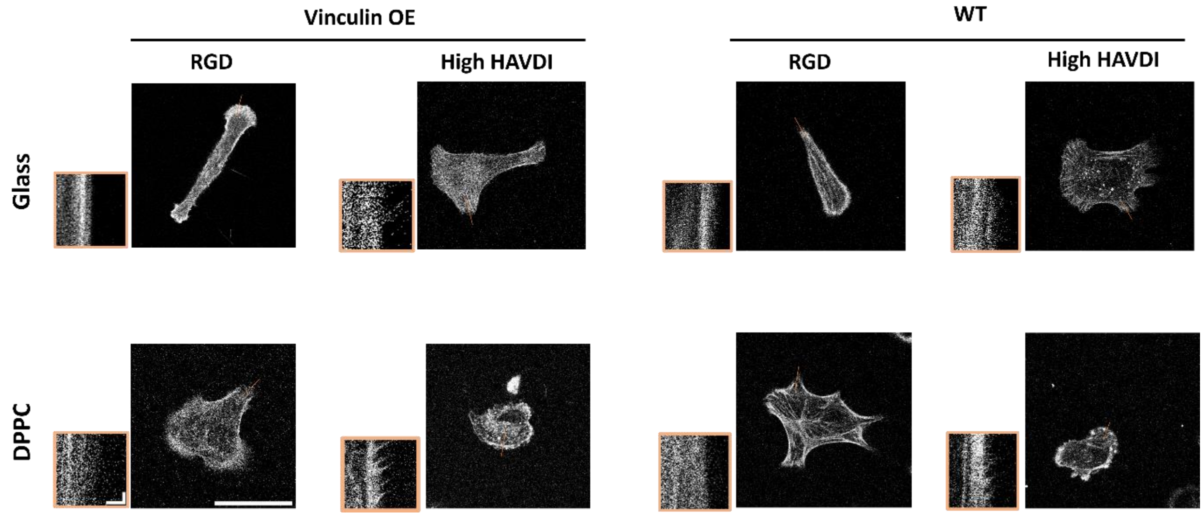

Supplementary Figure 14: Representative images of SPY555-FastAct actin probe in Y201 cells transfected to overexpress vinculin and their corresponding kymographs. Substrates include DPPC and glass functionalised with either RGD or RGD+HAVDI. Scale bars: cell images = 50 µm, kymographs= 5 µm horizontal and 30 s vertical.

Supplementary Fig. 15: Molecular clutch competition model predictions

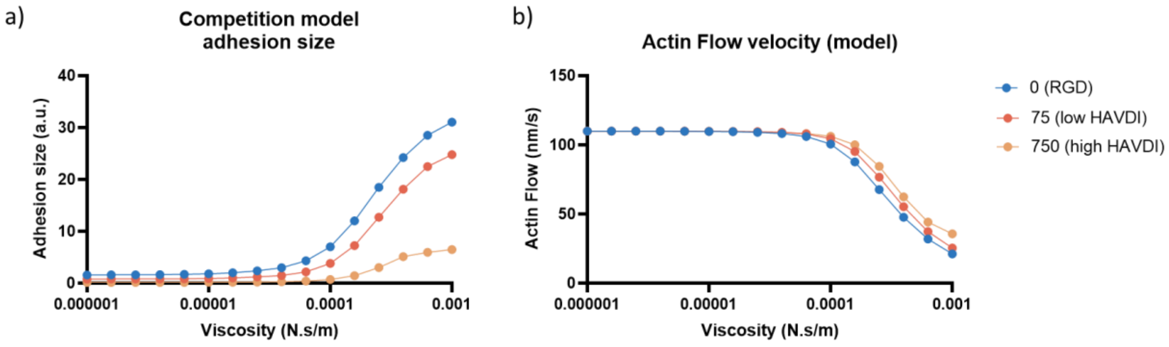

Supplementary Figure 15: a) Adhesion size obtained by applying the molecular clutch competition model. b) Theoretical actin flow velocity obtained by applying the competition in the molecular clutch model. Predictions are shown for different amounts of HAVDI ligands (0-75-750) at varying viscosity.

Supplementary note 1

Computational model implementation

To model cell adhesion formation and actin flows in response to viscosity and RGD/HAVDI ligands, we employed the same computational clutch model described previously[1], with the same parameters. The only modifications were the consideration of both RGD and HAVDI ligands, and the introduction of competition between them for actin binding.To model cell adhesion formation and actin flows in response to viscosity and RGD/HAVDI ligands, we employed the same computational clutch model described previously[1], with the same parameters. The only modifications were the consideration of both RGD and HAVDI ligands, and the introduction of competition between them for actin binding.

The model considers a given number of myosin motors (n_m) pulling on an actin bundle. This bundle can bind to the substrate through a set number of RGD molecules (n_{RGD}) and HAVDI molecules (n_{HAVDI}). Actin and RGD/HAVDI bind through integrins/cadherins and the different protein complexes that link integrins to actin, forming the "clutches" that transmit forces to the substrate. These clutches associate according to an effective binding rate (k_{onRGD} for RGD, and $k_{onHAVDI}$ for HAVDI) and dissociate according to unbinding rates k_{offRGD} or $k_{offHAVDI}$. These rates represent integrin-RGD or cadherin-HAVDI bonds. As the model considers a fixed number of ligands, the effective binding rate is given by

$$k_{on} = k_{ont} \dot{} d$$

where k_{ont} is the true binding rate characterizing each integrin-RGD bond (k_{ontRGD}) or cadherin-HAVDI bond ($k_{ontHAVDI}$), and is the density on the cell membrane of either integrins (d_{int}) or cadherins (d_{cad}). The unbinding rate (k_{offRGD} or $k_{offHAVDI}$) depends on the force applied to the bond, as observed experimentally both for integrin and cadherin bonds.

The Monte Carlo simulation begins with all ligand molecules unbound, in which case the myosin motors are unloaded and move the actin bundle with a constant rearward speed (towards the cell center) v_u. In each time step (5 ms), unbound ligands are allowed to associate with integrins/cadherins according to k_{on}, and bound molecules are allowed to dissociate according to k_{off}. In the next time step, resulting bound molecules move rearward with actin, become stretched, and apply a force to the substrate. All bound molecules are considered to be connected in parallel to the substrate, which is treated like a purely viscous substrate. After every time step the force applied to the substrate F_{sub} can be calculated by imposing force balance:

$$F_{sub} = \frac{v_u}{\frac{1}{n_{eng}\mu} + \frac{v_u}{n_m F_m}}$$

Where v_u is the contraction speed of myosin motors in the absence of force, μ is the viscosity of each ligand, n_{eng} is the number of engaged (bound) ligands, n_m is the total number of myosin motors, and F_m is the stall force of a single motor. This equation comes from combining our previously used equation to model a linear reduction in myosin contraction speed v with force:

$$v = v_u(1 - \frac{F_{sub}}{n_m F_m})$$

With the equation relating force to speed in a viscous system where each engaged ligand contributes with a given viscosity.

$$F_{sub} = n_{eng} v \mu$$

To implement reinforcement, the maximum force reached by each individual integrin (not cadherin) bond before unbinding is recorded. If this force is higher than a threshold reinforcement force F_r, integrin density is increases by a given amount d_{add}. Conversely, if the force is lower integrin density decreases by the same amount. This reflects the observation

that cell-matrix adhesion sites can both grow in response to force and shrink when forces are low. However, since initial integrin density d_{int} represents the amount of integrins before any significant adhesion growth, integrin density is not allowed to decrease below this value. Similarly, a maximum integrin density d_{max} is also set. Changes in d_{int} then affect the effective binding rate k_{on} according to eq. 1.

At the end of each step, the actin bundle moves according to v_a and displaces bound molecules by the same amount, increasing force and changing k_{off}. Bonds are then allowed to associate and dissociate according to the new k_{on} and k_{off}, and forces and velocities are recalculated. This process is repeated for 10000 s to ensure that steady state is reached.

<u>Competition between integrin and cadherin binding</u>

Integrin and cadherin both connect through adaptor proteins to actin, so we considered that they compete for binding to actin. To implement this as simply as possible, we simply considered that the probability of forming a successful clutch engaged to actin (modelled through binding rates) was proportional to the fraction of RGD or HAVDI clutches, with respect to the total amount of clutches. Thus, we corrected on-rates as:

$$k_{ontRGD}(corrected) = k_{ontRGD}\frac{n_{RGD}}{n_{RGD} + N_{HAVDI}}$$
$$k_{ontHAVDI}(corrected) = k_{ontHAVDI}\frac{n_{HAVDI}}{n_{RGD} + N_{HAVDI}}$$

Table 1. Model parameters.

All parameter values are the same as employed in our previous work[1], except the new parameters introduced to model cadherin bonds (shown in blue). Of note, the force-dependency of off-rates of cadherin bonds[2] has a very similar shape to that of α5β1 integrin bonds[3], but with a very different scale (over 100-fold higher). Thus, we modelled cadherin bonds with the same catch-bond dependency used for integrin bonds, but merely scaled them with a scaling factor.

Parameter	meaning	Value	Origin
n_m	Number of myosin motors	100	1
n_{RGD}	Number of RGD ligands	75	1
F_m	Myosin motor stall force	2 pN	1
v_u	Unloaded myosin motor velocity	110 nm/s	1
d_{int}	Initial integrin density on the membrane	300/μm^2	1
d_{intmax}	Maximum integrin density on the membrane	1000/μm^2	1
K_{ontRGD}	True integrin binding rate	1x10^{-4} μm^2/s	1
K_{offRGD}	Unbinding rate, scaling factor applied to force curve reported in[3]	0.5	1
$F_{threshold}$	Threshold reinforcement force	90 pN	1
d_{add}	Integrins added after each reinforcement event	6/μm^2	1
n_{HAVDI}	Number of HAVDI ligands	0-75-750	Adjusted
d_{cad}	Cadherin density on the membrane	300/μm^2	Adjusted
$k_{ontHAVDI}$	True cadherin binding rate	3.3x10^{-4} μm^2/s	4
$k_{offHAVDI}$	Unbinding rate, scaling factor applied to force curve reported in[3]	188	2

Table 2.

Statistical differences among early osteogenic differentiation markers of hMSCs seeded on DOPC, DPPC and Glass functionalized with RGD (0.2 % RGD) or low or high HAVDI (0.02 % or 2 % HAVDI respectively). *P ≤ 0.05, **P ≤ 0.01, ***P ≤ 0.001, ****P ≤ 0.0001.

**RUNX2 hMSCs statistical differences**			
DOPC :0.2 % RGD vs. DOPC :+ 0.02 % HAVDI	No	ns	0.9998
DOPC :0.2 % RGD vs. DOPC :+ 2 % HAVDI	No	ns	0.3614
DOPC :0.2 % RGD vs. DPPC :0.2 % RGD	No	ns	0.9923
DOPC :0.2 % RGD vs. DPPC :+ 0.02 % HAVDI	No	ns	0.6438
DOPC :0.2 % RGD vs. DPPC :+ 2 % HAVDI	No	ns	0.9961
DOPC :0.2 % RGD vs. Glass:0.2 % RGD	Yes	*	0.0126
DOPC :0.2 % RGD vs. Glass:+ 0.02 % HAVDI	Yes	**	0.0036
DOPC :0.2 % RGD vs. Glass:+ 2 % HAVDI	Yes	**	0.0018
DOPC :+ 0.02 % HAVDI vs. DOPC :+ 2 % HAVDI	No	ns	0.1976
DOPC :+ 0.02 % HAVDI vs. DPPC :0.2 % RGD	No	ns	>0.9999
DOPC :+ 0.02 % HAVDI vs. DPPC :+ 0.02 % HAVDI	No	ns	0.8457
DOPC :+ 0.02 % HAVDI vs. DPPC :+ 2 % HAVDI	No	ns	0.9996
DOPC :+ 0.02 % HAVDI vs. Glass:0.2 % RGD	No	ns	0.575
DOPC :+ 0.02 % HAVDI vs. Glass:+ 0.02 % HAVDI	No	ns	0.7015
DOPC :+ 0.02 % HAVDI vs. Glass:+ 2 % HAVDI	No	ns	0.1578
DOPC :+ 2 % HAVDI vs. DPPC :0.2 % RGD	No	ns	>0.9999
DOPC :+ 2 % HAVDI vs. DPPC :+ 0.02 % HAVDI	Yes	*	0.0207
DOPC :+ 2 % HAVDI vs. DPPC :+ 2 % HAVDI	No	ns	0.9995
DOPC :+ 2 % HAVDI vs. Glass:0.2 % RGD	No	ns	0.8389
DOPC :+ 2 % HAVDI vs. Glass:+ 0.02 % HAVDI	No	ns	0.9545
DOPC :+ 2 % HAVDI vs. Glass:+ 2 % HAVDI	No	ns	0.2142
DPPC :0.2 % RGD vs. DPPC :+ 0.02 % HAVDI	No	ns	0.8291
DPPC :0.2 % RGD vs. DPPC :+ 2 % HAVDI	No	ns	>0.9999
DPPC :0.2 % RGD vs. Glass:0.2 % RGD	No	ns	0.991
DPPC :0.2 % RGD vs. Glass:+ 0.02 % HAVDI	No	ns	0.9891
DPPC :0.2 % RGD vs. Glass:+ 2 % HAVDI	No	ns	0.8821
DPPC :+ 0.02 % HAVDI vs. DPPC :+ 2 % HAVDI	No	ns	0.8174
DPPC :+ 0.02 % HAVDI vs. Glass:0.2 % RGD	Yes	**	0.001
DPPC :+ 0.02 % HAVDI vs. Glass:+ 0.02 % HAVDI	Yes	**	0.0064
DPPC :+ 0.02 % HAVDI vs. Glass:+ 2 % HAVDI	Yes	***	0.0006
DPPC :+ 2 % HAVDI vs. Glass:0.2 % RGD	No	ns	0.9343
DPPC :+ 2 % HAVDI vs. Glass:+ 0.02 % HAVDI	No	ns	0.9231
DPPC :+ 2 % HAVDI vs. Glass:+ 2 % HAVDI	No	ns	0.1743
Glass:0.2 % RGD vs. Glass:+ 0.02 % HAVDI	No	ns	>0.9999
Glass:0.2 % RGD vs. Glass:+ 2 % HAVDI	No	ns	0.5967
Glass:+ 0.02 % HAVDI vs. Glass:+ 2 % HAVDI	No	ns	0.2485

Table 3.
Statistical differences among early chondrogenic differentiation markers of hMSCs seeded on DOPC, DPPC and Glass functionalized with RGD (0.2 % RGD) or low or high HAVDI (0.02 % or 2 % HAVDI respectively). *P ≤ 0.05, **P ≤ 0.01, ***P ≤ 0.001, ****P ≤ 0.0001.

**SOX9 hMSCs statistical differences**			
DOPC:0.2 % RGD vs. DOPC:+ 0.02 % HAVDI	No	ns	0.3327
DOPC:0.2 % RGD vs. DOPC:+ 2 % HAVDI	Yes	*	0.0138
DOPC:0.2 % RGD vs. DPPC:0.2 % RGD	No	ns	0.9968
DOPC:0.2 % RGD vs. DPPC:+ 0.02 % HAVDI	No	ns	0.4339
DOPC:0.2 % RGD vs. DPPC:+ 2 % HAVDI	Yes	**	0.0082
DOPC:0.2 % RGD vs. Glass:0.2 % RGD	Yes	*	0.0192
DOPC:0.2 % RGD vs. Glass:+ 0.02 % HAVDI	Yes	*	0.0238
DOPC:0.2 % RGD vs. Glass:+ 2 % HAVDI	No	ns	>0.9999
DOPC:+ 0.02 % HAVDI vs. DOPC:+ 2 % HAVDI	No	ns	0.0805
DOPC:+ 0.02 % HAVDI vs. DPPC:0.2 % RGD	No	ns	>0.9999
DOPC:+ 0.02 % HAVDI vs. DPPC:+ 0.02 % HAVDI	No	ns	0.9993
DOPC:+ 0.02 % HAVDI vs. DPPC:+ 2 % HAVDI	Yes	*	0.0102
DOPC:+ 0.02 % HAVDI vs. Glass:0.2 % RGD	Yes	**	0.0063
DOPC:+ 0.02 % HAVDI vs. Glass:+ 0.02 % HAVDI	No	ns	0.8125
DOPC:+ 0.02 % HAVDI vs. Glass:+ 2 % HAVDI	No	ns	0.8009
DOPC:+ 2 % HAVDI vs. DPPC:0.2 % RGD	No	ns	0.1525
DOPC:+ 2 % HAVDI vs. DPPC:+ 0.02 % HAVDI	Yes	*	0.0276
DOPC:+ 2 % HAVDI vs. DPPC:+ 2 % HAVDI	No	ns	0.9192
DOPC:+ 2 % HAVDI vs. Glass:0.2 % RGD	Yes	*	0.0125
DOPC:+ 2 % HAVDI vs. Glass:+ 0.02 % HAVDI	No	ns	0.4269
DOPC:+ 2 % HAVDI vs. Glass:+ 2 % HAVDI	Yes	*	0.0348
DPPC:0.2 % RGD vs. DPPC:+ 0.02 % HAVDI	No	ns	0.9992
DPPC:0.2 % RGD vs. DPPC:+ 2 % HAVDI	No	ns	0.198
DPPC:0.2 % RGD vs. Glass:0.2 % RGD	No	ns	0.0749
DPPC:0.2 % RGD vs. Glass:+ 0.02 % HAVDI	No	ns	0.7461
DPPC:0.2 % RGD vs. Glass:+ 2 % HAVDI	No	ns	0.9925
DPPC:+ 0.02 % HAVDI vs. DPPC:+ 2 % HAVDI	Yes	*	0.0107
DPPC:+ 0.02 % HAVDI vs. Glass:0.2 % RGD	Yes	*	0.0209
DPPC:+ 0.02 % HAVDI vs. Glass:+ 0.02 % HAVDI	No	ns	0.9695
DPPC:+ 0.02 % HAVDI vs. Glass:+ 2 % HAVDI	No	ns	0.6121
DPPC:+ 2 % HAVDI vs. Glass:0.2 % RGD	Yes	***	0.0006
DPPC:+ 2 % HAVDI vs. Glass:+ 0.02 % HAVDI	No	ns	0.5184
DPPC:+ 2 % HAVDI vs. Glass:+ 2 % HAVDI	Yes	*	0.0157
Glass:0.2 % RGD vs. Glass:+ 0.02 % HAVDI	Yes	**	0.0057
Glass:0.2 % RGD vs. Glass:+ 2 % HAVDI	No	ns	0.1738
Glass:+ 0.02 % HAVDI vs. Glass:+ 2 % HAVDI	No	ns	0.0739

Table 4.

Statistical differences among early adipogenic differentiation markers of hMSCs seeded on DOPC, DPPC and Glass functionalized with RGD (0.2 % RGD) or low or high HAVDI (0.02 % or 2 % HAVDI respectively). *P ≤ 0.05, **P ≤ 0.01, ***P ≤ 0.001, ****P ≤ 0.0001.

**PPAR hMSCs statistical differences**			
DOPC:0.2 RGD vs. DOPC:+ 0.02 HAVDI	No	ns	>0.9999
DOPC:0.2 RGD vs. DOPC:+ 2 HAVDI	No	ns	>0.9999
DOPC:0.2 RGD vs. DPPC:0.2 RGD	No	ns	0.8786
DOPC:0.2 RGD vs. DPPC:+0.02 HAVDI	No	ns	>0.9999
DOPC:0.2 RGD vs. DPPC:+ 2 HAVDI	No	ns	0.8502
DOPC:0.2 RGD vs. Glass:0.2 RGD	No	ns	0.5326
DOPC:0.2 RGD vs. Glass:+ 0.02 HAVDI	No	ns	0.4916
DOPC:0.2 RGD vs. Glass:+ 2 HAVDI	No	ns	0.8082
DOPC:0.2 RGD 0.02 HAVDI vs. DOPC:+ 2 HAVDI	No	ns	0.9978
DOPC:0.2 RGD 0.02 HAVDI vs. DPPC:0.2 RGD	No	ns	0.0632
DOPC:0.2 RGD 0.02 HAVDI vs. DPPC:+ 0.02 HAVDI	No	ns	>0.9999
DOPC:0.2 RGD 0.02 HAVDI vs. DPPC:+ 2 HAVDI	No	ns	0.5998
DOPC:0.2 RGD 0.02 HAVDI vs. Glass:0.2 RGD	No	ns	0.1719
DOPC:0.2 RGD 0.02 HAVDI vs. Glass:+ 0.02 HAVDI	No	ns	0.31
DOPC:0.2 RGD 0.02 HAVDI vs. Glass:+ 2 HAVDI	Yes	*	0.0185
DOPC:0.2 RGD 2 HAVDI vs. DPPC:0.2 RGD	No	ns	0.4504
DOPC:0.2 RGD 2 HAVDI vs. DPPC:+0.02 HAVDI	No	ns	0.9943
DOPC:0.2 RGD 2 HAVDI vs. DPPC:+ 2 HAVDI	No	ns	0.197
DOPC:0.2 RGD 2 HAVDI vs. Glass:0.2 RGD	No	ns	0.1097
DOPC:0.2 RGD 2 HAVDI vs. Glass:+ 0.02 HAVDI	No	ns	0.1325
DOPC:0.2 RGD 2 HAVDI vs. Glass:+ 2 HAVDI	No	ns	0.7482
DPPC:0.2 RGD vs. DPPC:+0.02 HAVDI	No	ns	0.6312
DPPC:0.2 RGD vs. DPPC:+ 2 HAVDI	No	ns	0.9896
DPPC:0.2 RGD vs. Glass:0.2 RGD	No	ns	0.4862
DPPC:0.2 RGD vs. Glass:+ 0.02 HAVDI	No	ns	>0.9999
DPPC:0.2 RGD vs. Glass:+2 HAVDI	No	ns	>0.9999
DPPC:0.2 RGD 0.02 HAVDI vs. DPPC:=2 HAVDI	No	ns	0.475
DPPC:0.2 RGD 0.02 HAVDI vs. Glass:0.2 RGD	No	ns	0.1902
DPPC:0.2 RGD 0.02 HAVDI vs. Glass:+ 0.02 HAVDI	No	ns	0.3187
DPPC:0.2 RGD 0.02 HAVDI vs. Glass:+ 2 HAVDI	No	ns	0.6243
DPPC:0.2 RGD 2 HAVDI vs. Glass:0.2 RGD	No	ns	0.0996
DPPC:0.2 RGD 2 HAVDI vs. Glass:+0.02 HAVDI	No	ns	0.3608
DPPC:0.2 RGD 2 HAVDI vs. Glass:+ 2 HAVDI	No	ns	>0.9999
Glass:0.2 RGD vs. Glass:+ 0.02 HAVDI	No	ns	0.8286
Glass:0.2 RGD vs. Glass:+ 2 HAVDI	No	ns	0.7475
Glass:0.2 RGD 0.02 HAVDI vs. Glass:+ 2 HAVDI	No	ns	0.9997

Table 5.
Statistical differences in focal adhesion length among Y201 non-transfected and transfected with talin, seeded on DOPC surfaces functionalized with RGD (0.2 % RGD) or low or high HAVDI (0.02 % or 2 % HAVDI respectively). Transfected conditions are indicated with a T. *P ≤ 0.05, **P ≤ 0.01, ***P ≤ 0.001, ****P ≤ 0.0001.

**DOPC Statistical differences talin transfection**			
0.2 % RGD vs. + low HAVDI	No	ns	0.9418
0.2 % RGD vs. + high HAVDI	No	ns	0.9967
0.2 % RGD vs. T 0.2 % RGD	No	ns	0.8967
0.2 % RGD vs. T + low HAVDI	No	ns	0.9973
0.2 % RGD vs. T + high HAVDI	No	ns	0.1153
+ low HAVDI vs. + high HAVDI	No	ns	0.9967
+ low HAVDI vs. T 0.2 % RGD	No	ns	0.4213
+ low HAVDI vs. T + low HAVDI	No	ns	0.821
+ low HAVDI vs. T + high HAVDI	Yes	*	0.0187
+ high HAVDI vs. T 0.2 % RGD	No	ns	0.6818
+ high HAVDI vs. T + low HAVDI	No	ns	0.9591
+ high HAVDI vs. T + high HAVDI	Yes	*	0.0469
T 0.2 % RGD vs. T + low HAVDI	No	ns	0.9967
T 0.2 % RGD vs. T + high HAVDI	No	ns	0.612
T + low HAVDI vs. T + high HAVDI	No	ns	0.3726

Table 6.
Statistical differences in focal adhesion length among Y201 non-transfected and transfected with talin, seeded on DPPC surfaces functionalized with RGD (0.2 % RGD) or low or high HAVDI (0.02 % or 2 % HAVDI respectively). Transfected conditions are indicated with a T. *P ≤ 0.05, **P ≤ 0.01, ***P ≤ 0.001, ****P ≤ 0.0001.

**DPPC Statistical differences talin transfection**			
0.2 % RGD vs. + 0.02 % HAVDI	No	ns	>0.9999
0.2 % RGD vs. + 2 % HAVDI	Yes	*	0.0402
0.2 % RGD vs. T 0.2 % RGD	No	ns	0.8127
0.2 % RGD vs. T + 0.02 % HAVDI	No	ns	0.0771
0.2 % RGD vs. T + 2 % HAVDI	No	ns	0.9249
+ 0.02 % HAVDI vs. + 2 % HAVDI	No	ns	0.857
+ 0.02 % HAVDI vs. T 0.2 % RGD	No	ns	0.3884
+ 0.02 % HAVDI vs. T + 0.02 % HAVDI	Yes	*	0.0219
+ 0.02 % HAVDI vs. T + 2 % HAVDI	No	ns	0.5384
+ 2 % HAVDI vs. T 0.2 % RGD	Yes	*	0.0108
+ 2 % HAVDI vs. T + 0.02 % HAVDI	Yes	****	<0.0001
+ 2 % HAVDI vs. T + 2 % HAVDI	Yes	*	0.0381
T 0.2 % RGD vs. T + 0.02 % HAVDI	No	ns	0.747
T 0.2 % RGD vs. T + 2 % HAVDI	No	ns	>0.9999
T + 0.02 % HAVDI vs. T + 2 % HAVDI	No	ns	0.6978

Table 7.
Statistical differences in focal adhesion length among Y201 non-transfected and transfected with talin, seeded on Glass surfaces functionalized with RGD (0.2 % RGD) or low or high HAVDI (0.02 % or 2 % HAVDI respectively). Transfected conditions are indicated with a T. *P ≤ 0.05, **P ≤ 0.01, ***P ≤ 0.001, ****P ≤ 0.0001.

**Glass Statistical differences talin transfection**			
0.2 % RGD vs. + 0.02 % HAVDI	No	ns	0.2454
0.2 % RGD vs. + 2 % HAVDI	Yes	****	<0.0001
0.2 % RGD vs. T 0.2 % RGD	No	ns	>0.9999
0.2 % RGD vs. T + 0.02 % HAVDI	No	ns	0.5849
0.2 % RGD vs. T + 2 % HAVDI	No	ns	>0.9999
+ 0.02 % HAVDI vs. + 2 % HAVDI	No	ns	0.2253
+ 0.02 % HAVDI vs. T 0.2 % RGD	No	ns	>0.9999
+ 0.02 % HAVDI vs. T + 0.02 % HAVDI	No	ns	>0.9999
+ 0.02 % HAVDI vs. T + 2 % HAVDI	No	ns	>0.9999
+ 2 % HAVDI vs. T 0.2 % RGD	Yes	***	0.0004
+ 2 % HAVDI vs. T + 0.02 % HAVDI	Yes	*	0.0226
+ 2 % HAVDI vs. T + 2 % HAVDI	Yes	***	0.0006
T 0.2 % RGD vs. T + 0.02 % HAVDI	No	ns	>0.9999
T 0.2 % RGD vs. T + 2 % HAVDI	No	ns	>0.9999
T + 0.02 % HAVDI vs. T + 2 % HAVDI	No	ns	>0.9999

Bibliography

1. Bennett, M. *et al.* Molecular clutch drives cell response to surface viscosity. *Proc. Natl. Acad. Sci. U.S.A.* **115**, 1192–1197 (2018).
2. Manibog, K., Li, H., Rakshit, S. & Sivasankar, S. Resolving the molecular mechanism of cadherin catch bond formation. *Nature Communications 2014 5:1* **5**, 1–11 (2014).
3. Kong, F., García, A. J., Mould, A. P., Humphries, M. J. & Zhu, C. Demonstration of catch bonds between an integrin and its ligand. *J Cell Biol* **185**, 1275–1284 (2009).
4. Leckband, D. & Sivasankar, S. Cadherin recognition and adhesion. *Curr Opin Cell Biol* **24**, 620–627 (2012).
