# Supplementary material for: N-cadherin crosstalk with integrin weakens the molecular clutch in response to surface viscosity
Source: Nat Commun. 2024 Oct 12;15:8824. doi: 10.1038/s41467-024-53107-6 (PMC11479646; doi:10.1038/s41467-024-53107-6)
Supplement: Supplementary file 2 — Reporting Summary [file 41467_2024_53107_MOESM2_ESM.pdf]

nature portfolio

Corresponding author(s): Manuel Salmeron

Last updated by author(s): Sep 22, 2024

Reporting Summary

Nature Portfolio wishes to improve the reproducibility of the work that we publish. This form provides structure for consistency and transparency in reporting. For further information on Nature Portfolio policies, see our Editorial Policies and the Editorial Policy Checklist.

Statistics

For all statistical analyses, confirm that the following items are present in the figure legend, table legend, main text, or Methods section.

n/a	Confirmed	
☐	☒	The exact sample size (*n*) for each experimental group/condition, given as a discrete number and unit of measurement
☐	☒	A statement on whether measurements were taken from distinct samples or whether the same sample was measured repeatedly
☐	☒	The statistical test(s) used AND whether they are one- or two-sided<br>*Only common tests should be described solely by name; describe more complex techniques in the Methods section.*
☐	☒	A description of all covariates tested
☐	☒	A description of any assumptions or corrections, such as tests of normality and adjustment for multiple comparisons
☐	☒	A full description of the statistical parameters including central tendency (e.g. means) or other basic estimates (e.g. regression coefficient) AND variation (e.g. standard deviation) or associated estimates of uncertainty (e.g. confidence intervals)
☐	☒	For null hypothesis testing, the test statistic (e.g. *F*, *t*, *r*) with confidence intervals, effect sizes, degrees of freedom and *P* value noted<br>*Give P values as exact values whenever suitable.*
☒	☐	For Bayesian analysis, information on the choice of priors and Markov chain Monte Carlo settings
☐	☒	For hierarchical and complex designs, identification of the appropriate level for tests and full reporting of outcomes
☒	☐	Estimates of effect sizes (e.g. Cohen's *d*, Pearson's *r*), indicating how they were calculated

Our web collection on statistics for biologists contains articles on many of the points above.

Software and code

Policy information about availability of computer code

Data collection	micromanager,PK Nanowizard 4 (JPK Instruments); ZEN, odyssey
Data analysis	Prism software v9 (GraphPad); JPK DP software, micromanager,, ZEN, odyssey

For manuscripts utilizing custom algorithms or software that are central to the research but not yet described in published literature, software must be made available to editors and reviewers. We strongly encourage code deposition in a community repository (e.g. GitHub). See the Nature Portfolio guidelines for submitting code & software for further information.

Data

Policy information about availability of data

All manuscripts must include a data availability statement. This statement should provide the following information, where applicable:

- Accession codes, unique identifiers, or web links for publicly available datasets
- A description of any restrictions on data availability
- For clinical datasets or third party data, please ensure that the statement adheres to our policy

All data supporting the findings in this study are available within the article and its Supplementary Information files, can be obtained from the corresponding author

Research involving human participants, their data, or biological material

Policy information about studies with human participants or human data. See also policy information about sex, gender (identity/presentation), and sexual orientation and race, ethnicity and racism.

Reporting on sex and gender	Cells used in this study are MSCs (https://promocell.com/product/human-mesenchymal-stem-cells-hmsc/) from Promocell and (Y201 MSCs developed at the University of York (https://pubmed.ncbi.nlm.nih.gov/26070611/)
Reporting on race, ethnicity, or other socially relevant groupings	n/a
Population characteristics	n/a
Recruitment	n/a
Ethics oversight	n/a

Note that full information on the approval of the study protocol must also be provided in the manuscript.

Field-specific reporting

Please select the one below that is the best fit for your research. If you are not sure, read the appropriate sections before making your selection.

[x] Life sciences [ ] Behavioural & social sciences [ ] Ecological, evolutionary & environmental sciences

For a reference copy of the document with all sections, see nature.com/documents/nr-reporting-summary-flat.pdf

Life sciences study design

All studies must disclose on these points even when the disclosure is negative.

Sample size	Sample size was determined on the basis of prior experience, conditions routinely included 3 or 4 replicates to ensure reproducibility and sufficient data points for statistical analysis (Cheng et al., 2018 , Donnelly et al., 2023., Ross et al., 2023, Sweeten 2019). 3 biological donors were tested for experiments . For each measurement more than 10 observations were made.
Data exclusions	No data were excluded from the analysis
Replication	Experiments were replicated independently 3 times with different donor cells, unless otherwise stated in the figure legends.
Randomization	Experimental conditions were assigned to culture wells without bias. Standard 24 well plates were used for cell culture experiments. Due to the nature of this research, randomization is not appropriate as assignment to categories must be exact therefore no systematic randomization was required.
Blinding	Blinding was not possible during this study as the same investigator was making the samples was the same measuring them.

Reporting for specific materials, systems and methods

We require information from authors about some types of materials, experimental systems and methods used in many studies. Here, indicate whether each material, system or method listed is relevant to your study. If you are not sure if a list item applies to your research, read the appropriate section before selecting a response.

Materials & experimental systems

n/a	Involved in the study	
[ ]	[x]	Antibodies
[ ]	[x]	Eukaryotic cell lines
[x]	[ ]	Palaeontology and archaeology
[x]	[ ]	Animals and other organisms
[x]	[ ]	Clinical data
[x]	[ ]	Dual use research of concern
[x]	[ ]	Plants

Methods

n/a	Involved in the study	
[x]	[ ]	ChIP-seq
[x]	[ ]	Flow cytometry
[x]	[ ]	MRI-based neuroimaging

Antibodies

Antibodies used	Anti-Vinculin: mouse monoclonal, sigma-Aldrich, V9263

anti P-FAK: mouse monoclonal, MilliPore, 05-1140
anti YAP: mouse monoclonal, Santa cruz biotechnology, sc-101199
anti N-Cadherin: Mouse monoclonal, BD biosciences,
anti integrin av: mouse monoclonal, Abcam, av11470
anti MIIA: rabbit monoclonal, Abcam,
anti RUNX2: mouse monoclonal, Abgen
anti P-RUNX2: rabbit monoclonal, Arigo biolaboratories, arg57989
anti SOX9: mouse monoclonal, Santa cruz biotechnology,
anti PPAR: mouse monoclonal, Santa cruz biotechnology
anti beta-catenin: mouse monoclonal, abcam

Validation

Describe the validation of each primary antibody for the species and application, noting any validation statements on the manufacturer's website, relevant citations, antibody profiles in online databases, or data provided in the manuscript.

Eukaryotic cell lines

Policy information about cell lines and Sex and Gender in Research

Cell line source(s)

Bone marrow human mesenchymal stem cells were sourced by promocell.
Y201 cells were sourced from Paul Genever's group in university of York

Authentication

None of these cell lines were authenticated

Mycoplasma contamination

All cells tested negative for microplasma contamination

Commonly misidentified lines
(See ICLAC register)

NA

Plants

Seed stocks

Report on the source of all seed stocks or other plant material used. If applicable, state the seed stock centre and catalogue number. If plant specimens were collected from the field, describe the collection location, date and sampling procedures.

Novel plant genotypes

Describe the methods by which all novel plant genotypes were produced. This includes those generated by transgenic approaches, gene editing, chemical/radiation-based mutagenesis and hybridization. For transgenic lines, describe the transformation method, the number of independent lines analyzed and the generation upon which experiments were performed. For gene-edited lines, describe the editor used, the endogenous sequence targeted for editing, the targeting guide RNA sequence (if applicable) and how the editor was applied.

Authentication

Describe any authentication procedures for each seed stock used or novel genotype generated. Describe any experiments used to assess the effect of a mutation and, where applicable, how potential secondary effects (e.g. second site T-DNA insertions, mosiacism, off-target gene editing) were examined.
